# Changes in carnitine levels through induction chemotherapy in head and neck cancer patients as a potential cause of therapy-related malaise

**DOI:** 10.1186/s12885-021-08471-7

**Published:** 2021-06-28

**Authors:** Tatsuya Ito, Kiyoaki Tsukahara, Hiroki Sato, Akira Shimizu, Isaku Okamoto

**Affiliations:** Department of Otorhinolaryngology, Head and Neck Surgery, Tokyo Medical University, 6-7-1 Nishishinjyuku, Shinjyuku-ku, Tokyo, Japan

**Keywords:** Carnitine, Free carnitine, Induction chemotherapy, Head and neck cancer, Cisplatin

## Abstract

**Background:**

Carnitine is related to malaise, and cisplatin is associated with decreased carnitine. The purpose of this study was to elucidate the effects of one course of induction chemotherapy (IC) for head and neck cancer on blood carnitine levels, focusing on free carnitine (FC).

**Methods:**

This single-center prospective study investigated 20 patients diagnosed with primary head and neck cancer who underwent IC with cisplatin, docetaxel, and 5-fluorouracil. FC, acylcarnitine (AC), and total carnitine (TC) levels were measured before starting therapy and on Days 7 and 21 after starting IC. In addition, malaise was evaluated before and after therapy using a visual analog scale (VAS).

**Results:**

All subjects were men and the most common primary cancer site was the hypopharynx (9 patients). FC levels before starting therapy and on Days 7 and 21 were 47.7 ± 2.2 μM/mL, 56.7 ± 2.2 μM/mL, and 41.1 ± 1.9 μM/mL, respectively. Compared with the baseline before starting therapy, FC had significantly decreased on Day 21 (*p* = 0.007). AC levels before starting therapy and on Days 7 and 21 were 12.5 ± 1.2 μM/mL, 13.6 ± 1.4 μM/mL, and 10.7 ± 0.7 μM/mL, respectively. TC levels before starting therapy and on Days 7 and 21 were 60.2 ± 2.5 μM/mL, 70.2 ± 3.3 μM/mL, and 51.7 ± 2.3 μM/mL, respectively. No significant differences in AC, TC or VAS were seen before the start of therapy and on Day 21.

**Conclusions:**

After IC, a latent decrease in FC occurred without any absolute deficiency or subjective malaise.

## Introduction

Carnitine is a metabolic substance involved in fat metabolism. Absolute deficiency is defined as free carnitine (FC) < 36 μM/mL in the Guidelines for Diagnosis and Treatment of Carnitine Deficiency 2018 [1], so blood kinetics of FC are important. Vinci et al. described free carnitine concentrations of 41.9 ± 8.4 μM/mL in a healthy population, 39.5 ± 7.8 μM/mL in cancer patients, and 33.7 ± 9.4 μM/mL in cachexic patients. Levels are known to be lower in patients with cachexia than in patients without cachexia [2]. Cancer patients are thought to have reduced carnitine levels due to reduced dietary intake and impaired endogenous synthesis [3]. The distribution of carnitine is known to be related to organic cation transporter (OCTN), ATB^0,+^, OAT9, and other proteins. OCTN2 is involved mainly in carnitine transport [4], and this sodium-dependent carnitine transporter is seen in nearly all tissues in the body [5–8]. Almost all carnitine is filtered into the urine via the renal glomeruli, then reabsorbed into the body by OCTN2 expressed in the renal tubules [9–11]. Cisplatin is a key drug in head and neck cancer treatments and is used in a variety of cases, such as induction chemotherapy (IC) and concurrent chemoradiotherapy (CCRT). Cisplatin blocks OCTN2 and inhibits the expression of OCTN2 distributed in tubular cells due to nephrotoxicity [12]. Cisplatin is thus an anticancer agent that increases urinary excretion of carnitine and reduces expression of OCTN2, causing carnitine deficiency. Carnitine deficiency in turn leads to malaise, which is a common reason for discontinuing cancer therapy [13]. Elucidation of how IC affects blood carnitine kinetics thus has important implications. However, no reports to date have examined associations between IC for head and neck cancer and changes in blood carnitine levels. The purpose of this study was to elucidate the effects of one course of IC for head and neck cancer on blood carnitine levels, focusing on FC.

## Materials and methods

### Patients and ethics

Subjects were 20 patients ≥20 but < 75 years old with stage III or IV-A head and neck cancer who underwent IC as the first treatment. Eastern Cooperative Oncology Group performance status (PS) was 0–2. Exclusion criteria were: administration of levocarnitine within the past month or current prescription of levocarnitine; pregnant or breastfeeding state, or possible pregnancy for women; or being considered as unsuitable for the trial by the patient’s primary care physician. The enrollment period was from August 2, 2016 to December 31, 2019. Written consent for participation in the trial was obtained from all patients prior to enrolment. This single-center, prospective observational study was approved by the Research Ethics Committee of Tokyo Medical University Hospital (approval no. 2016–058).

### Assessments

The primary endpoint was serum carnitine level before and after IC. Total carnitine (TC) and FC were measured from collected blood. Direct measurement of acylcarnitine (AC) was difficult, so AC level was calculated as “TC - FC”, based on the notion that “TC = FC + AC”. Serum carnitine was measured before IC (Pre-IC), 7 days after starting IC (Post-IC7), and 21 days after starting IC (Post-IC21). In accordance with the Guidelines for Diagnosis and Treatment of Carnitine Deficiency 2018 [1], FC < 36 μM/mL was taken as representing absolute deficiency and AC/FC < 0.4 as relative deficiency. The secondary endpoint was subjective malaise assessed at Pre-IC, Post-IC7, and Post-IC21 using a visual analog scale (VAS). The VAS had a total length of 70 mm, scored in 1-mm increments, with 0 mm indicating no malaise and 70 mm indicating severe malaise. Staging was performed according to the Union for International Cancer Control (UICC) TNM Classification of Malignant Tumors, 7th edition. Computed tomography (CT) was performed between 21 and 28 days after starting IC, and therapeutic effects were evaluated by a radiological specialist according to Response Evaluation Criteria in Solid Tumors (RECIST) version 1.1 guidelines.

### Chemotherapy regimen

As IC, cisplatin and docetaxel were intravenously infused at a dose of 60 mg/m^2^ each on day 1 and 5-fluorouracil was intravenously infused at a dose of 600 mg/m^2^ on days 1–5 for 24 h.

### Statistical analysis

Tests of normality were performed for concentrations of FC, AC, and TC, and for VAS score. When a normal distribution was identified, comparisons were made using repeated-measures analysis of variance. Data that did not follow a normal distribution were tested with the Friedman test. Items showing a significant difference were tested using the Bonferroni method. Statistical analysis was performed using IBM® SPSS® Statistics 26 (IBM Corp., Tokyo, Japan). All tests were two-sided, with values of *p* < 0.05 taken to indicate a significant difference.

## Results

### Patient characteristics

Patient characteristics are shown in Table 1. All patients were men, with a median age of 64 years (range, 48–73 years). The primary site was the oropharynx in 8 patients, hypopharynx in 9 patients, larynx in 2 patients, and maxillary sinus in 1 patient. Tumor stage was Stage III in 4 patients and Stage IV in 16 patients. The therapeutic effect was complete response (CR) in 1 patient, partial response (PR) in 14 patients, stable disease (SD) in 4 patient, and progressive disease (PD) in 1 patient.

**Table 1:** Patient characteristics

Characteristic	*N*=20
Age, years (median)	48-73 (64)
Sex	Male:20 cases Female:0 cases
Primary resion	Oropharynx: 8 cases
	Hypopharynx: 9 cases
	Larynx: 2 cases
	Maxillary sinus: 1 case
Stage	III: 4 cases IV: 16 cases
Therapeutic effect	Complete response: 1 case Partial response: 14 cases Stable response: 4 cases Progressive disease: 1 case

### Changes in FC, AC, and TC

**Fig. 1:**
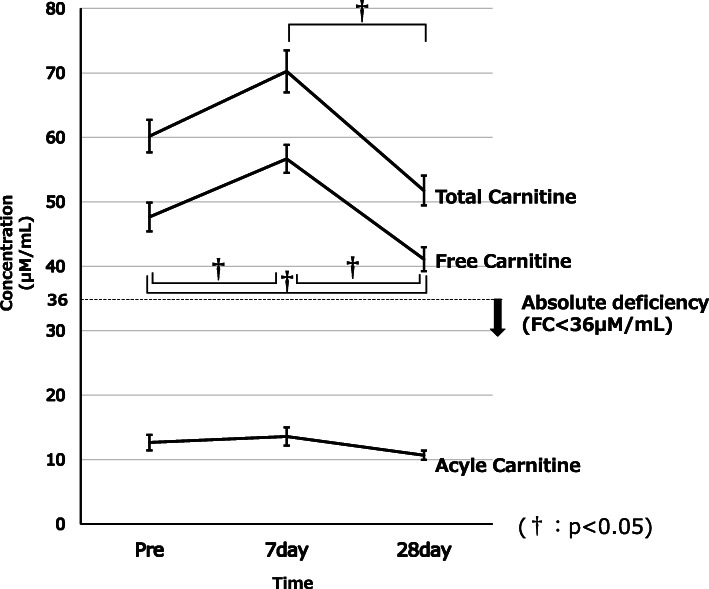
Changes in carnitine values. Compared with Pre-IC, FC was significantly decreased on Post-IC21. However, absolute deficiency did not develop

Changes in carnitine are shown in Fig. 1. FC levels at Pre-IC, Post-IC7, and Post-IC21 were 47.7 ± 2.2 μM/mL, 56.7 ± 2.2 μM/mL, and 41.1 ± 1.9 μM/mL, respectively. FC was significantly lower at Post-IC21 than at Pre-IC (*p* = 0.007), but significantly higher at Post-IC7 than at Pre-IC (*p* = 0.001). FC level was significantly lower at Post-IC21 than at Post-IC7 (*p* = 0.0001). Absolute deficiency (FC < 36 μM/mL) was identified in 3 patients (15%) at Pre-IC, 1 patient (5%) at Post-IC7, and 1 patient (5%) at Post-IC21.

AC levels at Pre-IC, Post-IC7, and Post-IC21 were 12.5 ± 1.2 μM/mL, 13.6 ± 1.4 μM/mL, and 10.7 ± 0.7 μM/mL, respectively. No significant differences were seen between any time points.

TC levels at Pre-IC, Post-IC7, and Post-IC21 were 60.2 ± 2.5 μM/mL, 70.2 ± 3.3 μM/mL, and 51.7 ± 2.3 μM/mL, respectively. Levels tended to be lower at Pre-IC and Post-IC21, but no significant differences were seen (*p* = 0.09). No significant difference was seen between Pre-IC and Post-IC7 (*p* = 0.48). In contrast, TC was significantly lower at Post-IC21 than at Post-IC7 (*p* = 0.01).

### Changes in AC/FC ratio

**Fig. 2:**
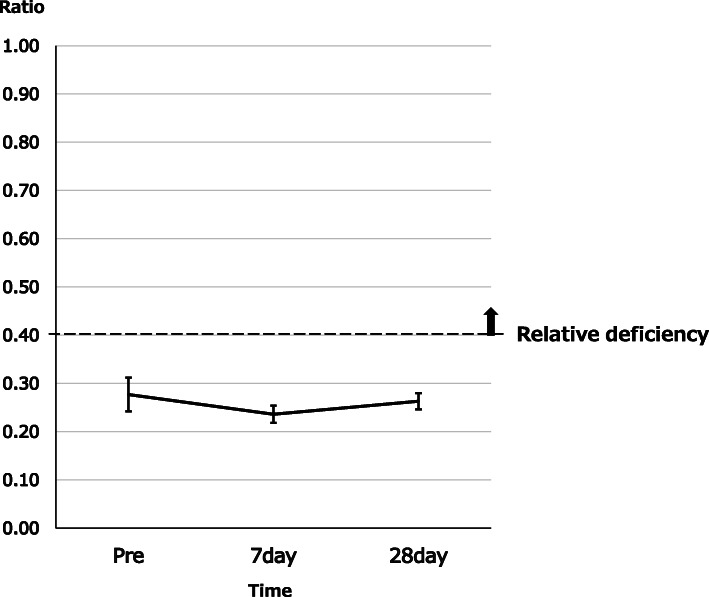
Changes in AC/FC ratio. No significant differences were seen between any times, and no relative deficiencies were identified

Changes in AC/FC ratio are shown in Fig. 2. Ratios at Pre-IC, Post-IC7, and Post-IC21 were 0.28 ± 0.04, 0.24 ± 0.02, and 0.26 ± 0.02, respectively. No significant differences were seen between any time points. Relative carnitine deficiency (AC/FC ratio < 0.4) was seen in 3 patients (15%) at Pre-IC and 4 patients (20%) at Post-IC21. No patients showed relative carnitine deficiency at Post-IC7.

### Changes in VAS

**Fig. 3:**
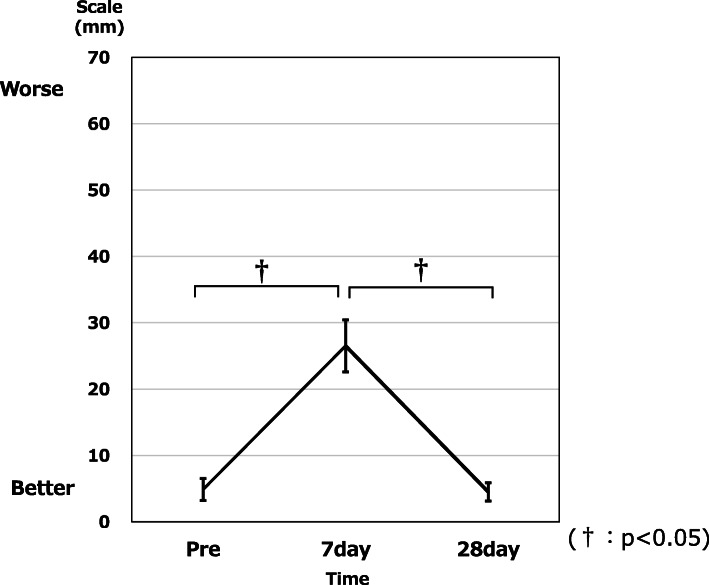
Changes in VAS. A significant worsening was identified on Post-IC7. Scores were about the same at Pre-IC and Post-IC21

Changes in VAS score are shown in Fig. 3. No significant difference was seen between Pre-IC and Post-IC21 (*p* = 0.949). VAS score was significantly higher at Post-IC7 than at Pre-IC (*p* = 0.000232), and significantly lower at Post-IC21 than at Post-IC7 (*p* = 0.000467). No significant difference in VAS was seen between patients with absolute carnitine deficiency (3 cases) and normal patients (17 cases) (*p* > 0.05).

## Discussion

This study investigated changes in blood carnitine levels, including FC, in patients who underwent IC for head and neck cancer. FC levels were significantly lower at Post-IC21 than at Pre-IC. If 2–3 courses of IC were to be performed, FC levels would presumably be even lower. At the same time, absolute carnitine deficiency was seen in 1 patient (5%) and relative carnitine deficiency was seen in 4 patients (20%) at Post-IC21. Although FC was significantly decreased compared with before the start of therapy, few patients reached absolute or relative carnitine deficiency. VAS scores in this study were thus also returned to nearly Pre-IC levels by Post-IC21. This is probably one reason why attention has not been focused on blood carnitine kinetics during IC.

Around 75% of the carnitine content in the body is consumed orally. The rest is biosynthesized in the cardiac muscle, kidneys, and brain [9–11]. The main causes of carnitine deficiency are thus insufficient dietary intake and decreased muscle mass. Muscle mass is known to decrease in cancer patients. Patients with head and neck cancer show a tendency for an unbalanced diet accompanied by high alcohol consumption and dysphagia due to pharyngeal tumors. Against this background, malnutrition from decreased dietary intake is seen in 25–50% of patients [14–16]. Despite advances in supportive therapy, dietary intake falls even further when cisplatin is used due to nausea and other adverse effects [17]. In terms of the energy metabolism of cancer cells, a change occurs from aerobic metabolism of fatty acids to anaerobic metabolism with glycolysis. This is called the Warburg effect, in which AC is increased and FC is decreased due to incomplete β oxidation, increasing the AC/FC ratio [18]. Among our patients, absolute carnitine deficiency was seen in 3 patients (15%) and relative carnitine deficiency in 3 patients (15%) before the start of IC. Carnitine deficiency thus appears to occur easily in head and neck cancer patients both before and during treatment. On the other hand, elevated blood carnitine levels were seen on Post-IC7 in our patients. Blood carnitine levels are reportedly temporarily elevated with the administration of anticancer agents [19–21]. Unfortunately, the underlying physiological reasons have yet to be clarified. One possibility suggested in the literature is that migration of FC into cells is blocked and blood FC is temporarily elevated when anticancer agents block the OCTN2 expressed on cell membranes [22]. Despite the significant elevation of FC on Post-IC7 in our patients, malaise worsened on the VAS. This is attributed to FC in blood not being efficiently used in energy production, supporting the hypothesis that FC migration into cells is blocked when anticancer agents block OCTN2. Physiological elucidation of carnitine blood kinetics is awaited.

In this study, a significant decrease in FC was shown after one course of IC. That is, one course of IC achieved a latent decrease in FC, even though no absolute carnitine deficiency developed and VAS score also improved to the same level as Pre-IC. Evaluations in this study were performed using a VAS that allows easy comparison before and after treatment with a single item. Other multi-item evaluation methods are in current clinical use, such as the Cancer Fatigue Scale (CFS) and the Daily Fatigue Cancer Scale [23, 24]. The VAS was used in this study as a familiar method to evaluate various subjective experiences due to its wide score range and high sensitivity [25]. In the future, if the number of cases increases, we will consider methods such as the CFS and Daily Fatigue Cancer Scale.

## Conclusion

This study investigated the effects of IC for head and neck cancer on blood carnitine kinetics, including FC. FC was significantly decreased at Post-IC21 compared with Pre-IC. After IC, FC was in a state of latent decrease.

## Data Availability

The datasets used and/or analyzed during the current study are available from the corresponding author on reasonable request.
